# Sequence analysis and structure prediction of ABHD16A and the roles of the ABHD family members in human disease

**DOI:** 10.1098/rsob.180017

**Published:** 2018-05-23

**Authors:** Jun Xu, Weizhen Gu, Kai Ji, Zhao Xu, Haihua Zhu, Wenming Zheng

**Affiliations:** 1 College of Life Sciences, Henan Agricultural University, 95 Wenhua Road, Zhengzhou 450002, People's Republic of China; 2 Henan Business Research Institute Co. Ltd, Zhengzhou, He'nan, People's Republic of China

**Keywords:** ABHD16A, ABHDs, metabolic disease, lipase, immune regulation

## Abstract

Abhydrolase domain containing 16A (ABHD16A) is a member of the α/β hydrolase domain-containing (ABHD) protein family and is expressed in a variety of animal cells. Studies have shown that ABHD16A has acylglycerol lipase and phosphatidylserine lipase activities. Its gene location in the main histocompatibility complex (MHC) III gene cluster suggests that this protein may participate in the immunomodulation of the body. The results of studies investigating nearly 20 species of ABHDs reveal that the ABHD proteins are key factors in metabolic regulation and disease occurrence and development. In this paper, we summarize the related progress regarding the function of ABHD16A and other ABHD proteins. A prediction of the active sites and structural domains of ABHD16A and an analysis of the amino acid sites are included. Moreover, we analysed the amino acid sequences of the ABHD16A molecules in different species and provide an overview of the related functions and diseases associated with these proteins. The functions and diseases related to ABHD are systematically summarized and highlighted. Future research directions for studies investigating the functions and mechanisms of these proteins are also suggested. Further studies investigating the function of ABHD proteins may further confirm their positions as important determinants of lipid metabolism and related diseases.

## 1. Introduction

Human adhydrolase domain containing 16A (ABHD16A) is a 63 kDa protein containing 558 amino acid residues that is expressed in cells in multiple species. ABHD16A is a serine metabolism enzyme that typically contains an α/β hydrolase domain. Recently, studies have shown that ABHD16A is associated with neurodegenerative disease [1], immunoregulation [2], Kawasaki disease and coronary artery aneurysm [3]. Human ABHD16A is also known as human leucocyte antigen B (HLA-B) associated transcript 5 (BAT5). Because of its conserved features, ABHD was first identified in 1992 [4] and considered among the most diverse and widespread protein families, including esterases, proteases, lipases, peroxidases, epoxide hydrolases and dehalogenases [5]. Mammalian ABHDs, which are hydrolases, participate in lipid metabolism, intracellular signalling transduction and metabolic disorders [6]. Particularly within the prior 2–3 years, several groups have reported that ABHD2 [7], ABHD6 [8], ABHD12 [9], ABHD16 [2] and ABHD17 [10] could function in inflammation regulation and cancer pathogenesis. Although functional studies investigating the ABHDs are limited and the field is in its infancy, the growing awareness of the biological significance of the ABHDs has stimulated research in this field. Here, we discuss the current research state of ABHD16A, including its gene location and related functions. We also analysed the amino acid sequences and constructed a phylogeny tree of ABHD16A. The functions of other ABHD proteins are systematically summarized and discussed. Significant insights and future developments are also proposed.

## 2. Gene location of ABHD16A

**Figure 1.**
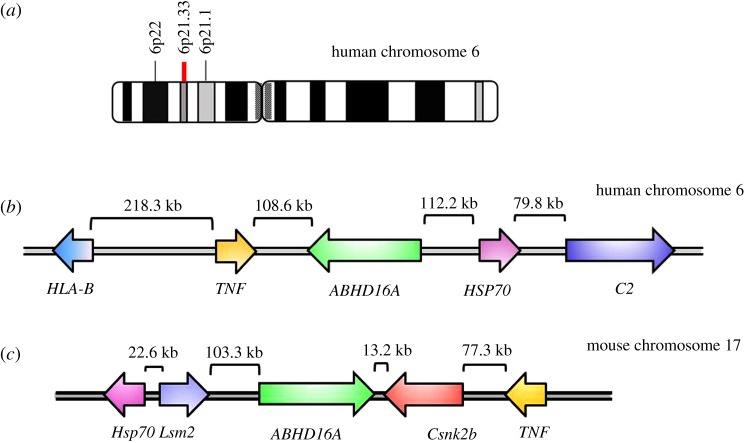
Position of the ABHD16A gene. Chromosome position of the human ABHD16A gene (*a*). ABHD16A is conserved in humans (*b*) and mice (*c*), and, together with other immunity-related genes, forms a gene cluster in the MHC III region. The coloured arrows indicate different genes and their transcriptional directions. The base pair numbers shown on the horizontal line indicate the distance between the genes.

Human ABHD16A is located on chromosome 6p21.33. This gene has 21 exons and four different transcripts, two of which encode proteins (NM_001177515.1 and NM_021160.2), while the other two transcripts encode long non-coding RNAs (NR_033488.1 and NR_033489.1). The *Abhd16a* and *bat2-bat5* genes are closely associated with tumour necrosis factor (TNF) and the complement gene cluster C2 genes (figure 1*a*,*b*), and are located within the human major histocompatibility complex class III (MHC III) region [11–13]. Homoplastically, the mouse ABHD16A gene is located between TNF and Heat shock protein 70 (HSP70) near the Ck2b protein kinase gene (figure 1*c*) in the cluster of MHC III [13–15]. Owing to the position characteristics of the ABHD16A gene described above, the BAT1–BAT5 proteins have been predicted to be associated with some aspects of immunity.

## 3. Protein structure of ABHD16A

**Figure 2.**
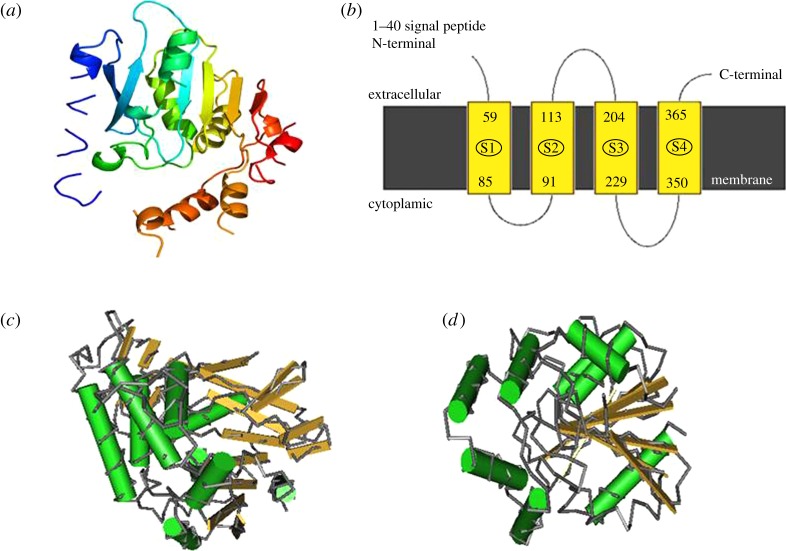
Structural prediction of the human ABHD16A protein. (*a*) Three-dimensional patterns predicted by Phyre2. (*b*) Transmembrane region predicted by Phyre2. (*c*) Abhydrolase onefold prediction by the Conserved Domains Database of NCBI. (*d*) BioH domain predicted by the Conserved Domains Database of NCBI.

We submitted the amino acid sequence of human ABHD16A to the Phyre2 portal to predict its three-dimensional protein structure (figure 2*a*) and transmembrane region (figure 2*b*). The results revealed four transmembrane regions: residues 59–85, 91–113, 204–229 and 350–365. The sequence alignment against the BLAST and Conserved Domains Database of NCBI revealed that ABHD16A has three conserved domains similar to Abhydrolase 1 (figure 2*c*), BioH (figure 2*d*) and PldB (Phospholipase D-orthologue B). The Abhydrolase 1 domain comprises amino acid residues 280 to 408. Many hydrolytic enzymes possess this catalytic domain. These enzymes conservatively preserve the catalytic residues, but not the binding sites, from their common ancestor [4]. BioH was identified as a biotin synthesis enzyme and was predicted to contribute to fatty acid synthesis because of its carboxylesterase activity in substrates with short acyl chains [16,17]. The BioH domain of ABHD16A is located in amino acids 276–428, but its precise function must be further investigated in greater detail. The PldB domain has a 3D structure similar to that of Abhydrolase 1, is located within amino acid residues 302–398 and exhibits lysophospholipase activity [18–20].

The predicted protein structure of ABHD16A is similar to that of other ABHDs. Approximately 23 different ABHD proteins belonging to the α/β-hydrolase-fold superfamily have been reported thus far. A typical α/β-hydrolase fold has 8 β-strands and 6 α-helices [6]. The hydrolytic enzyme active centre is formed by histidine residues and surrounded by helices and loops linking the β-strands. In most cases, Ser and occasionally Cys or Asp lie in a compact loop. In addition, a highly conserved histidine residue is present in a variable loop behind β8 [6].

## 4. Conservation analysis of the amino acid sequence of ABHD16A

**Figure 3.**
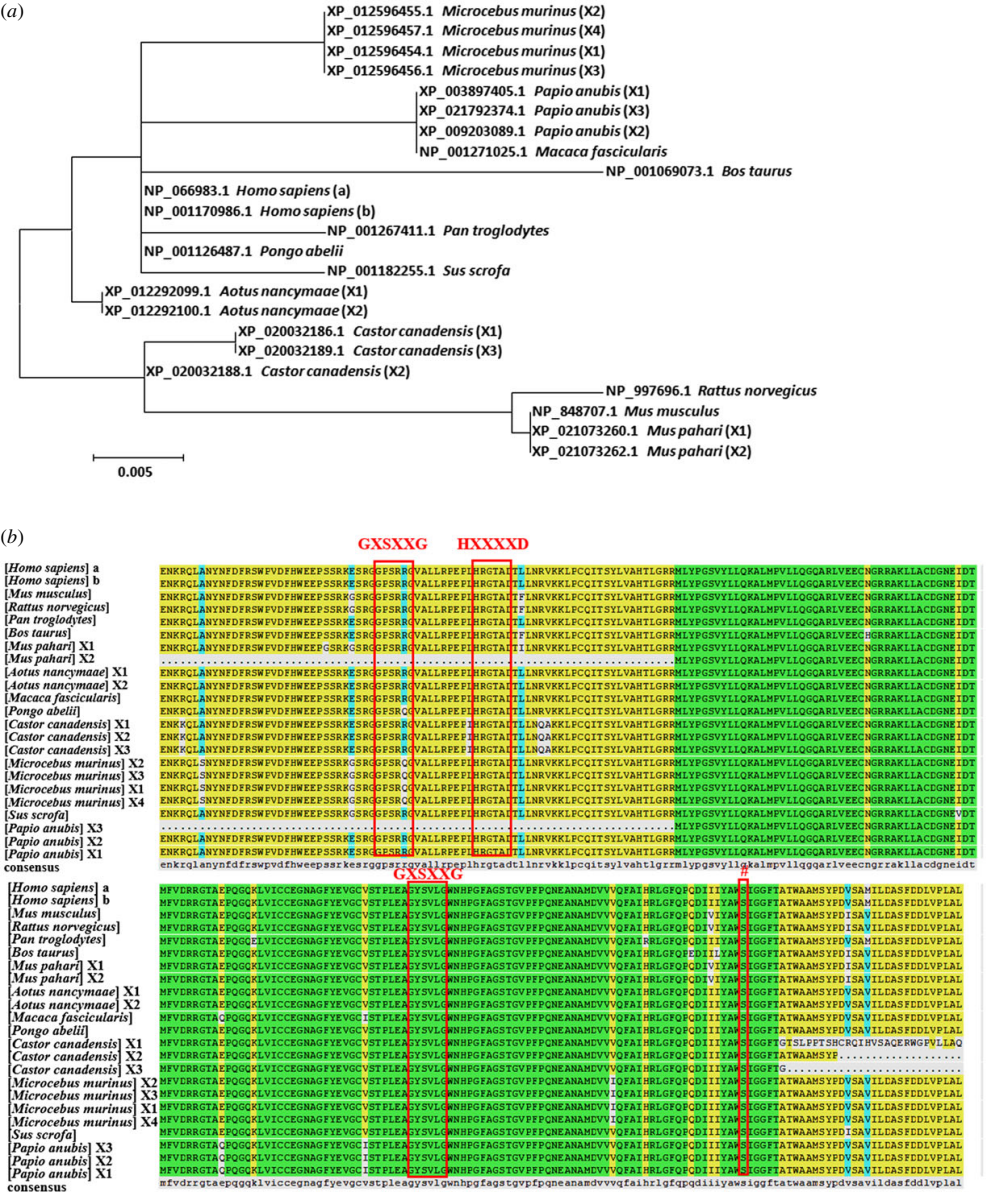
Conservation analysis of the amino acid sequence of ABHD16A. (*a*) Phylogenetic tree of the ABHD16A amino acid sequences from 13 mammalian species. Codes prefixed by X represent different variants. The phylogenetic tree was constructed using the neighbour-joining method by MEGA. (*b*) Multiple alignment of ABHD16A in 13 mammals using DNAMAN, and the different colours represent different homologies of amino acids. The amino acid residues in the red boxes indicate the predicted instructions of the lipase-like motif (GXSXXG), the conserved (HXXXXD) motif and the active nucleophile centre (#, Ser, Cys or Asp), respectively.

ABHD16A is a highly conserved protein in mammals that is expressed in cells in different tissues [19]. Using molecular evolutionary genetics analysis (MEGA) software, we analysed the amino acid sequences of ABHD16A in 13 mammalian species (table 1). The results revealed 412 conserved sites, 146 variable sites, 58 parsimony-informative sites and 88 singleton sites. Both the maximum-likelihood phylogenetic analysis and sequence comparison showed that the ABHD16A protein sequences could be divided into three categories (figure 3*a*). The significant differences in the length of the ABHD16A polypeptide chain imply that variable splicing occurs during the post-transcriptional processing of ABHD16A mRNA. For example, human isoforms a and b of ABHD16A have 558 and 525 aa, respectively; Sikkim mice isoforms a and b have 558 and 339 aa, respectively. However, the functional domains, including the alpha/beta hydrolytic enzyme domain, acyltransferase motif HXXXXD (H, histidine; D, aspartic acid and X, any residues), lipase-like motifs GXSXXG (G, glycine; S, serine and X, any residues) and nucleophile centres (Ser, Cys or Asp), are highly conserved (figure 3*b*) [6]. The present analysis further confirmed the findings of previous reports [6,19] and that ABHD16A is genetically distant from the other members of the ABHD family [6].

**Table 1.** References of ABHD16A in 13 mammalian species.

species	GenBank GI no.
*Homo sapiens*	NP_066983.1(isoform a), NP_001170986.1(isoform b)
*Mus musculus*	NP_848707.1
*Rattus norvegicus*	NP_997696.1
*Pan troglodytes*	NP_001267411.1
*Bos taurus*	NP_001069073.1
*Microcebus murinus*	XP_012596454.1(isoform X1), XP_012596455.1(isoform X2), XP_012596456.1(isoform X3), XP_012596457.1(isoform X4)
*Mus pahari*	XP_021073260.1(isoform X1), XP_021073262.1(isoform X2)
*Castor canadensis*	XP_020032186.1(isoformX1), XP_020032188.1(isoform X2), XP_020032189.1(isoform X3)
*Papio anubis*	XP_003897405.1(isoform X1), XP_009203089.1(isoform X2), XP_021792374.1(isoform X3)
*Sus scrofa*	NP_001182255.1
*Aotus nancymaae*	XP_012292099.1(isoform X1), XP_012292100.1(isoform X2)
*Macaca fascicularis*	NP_001271025.1
*Pongo abelii*	NP_001126487.1

## 5. Research progress related to ABHD16A

In 1989, Spies *et al*. [12] predicted that ABHD16 probably functioned in immune regulation processes because its gene location is similar to that of TNF and HSP70 in the MHC III gene cluster [12,13]. However, during the following two decades, few gratifying results regarding ABHD16A were reported. Subsequently, Mathew's group reported that the expression of ABHD16A could influence the immunogenicity of bone marrow cells in recombinant B10.BR mice [21]. By analysing the polymorphisms and haplotypes of ABHD16A, Hsieh *et al.* [3] found several associations between ABHD16A and the genetic predisposition to coronary artery aneurysm and Kawasaki disease. However, during the following few years, the underlying mechanism was not further explored.

In 2014, Savinainen *et al*. pioneered a study investigating the enzymatic characteristics of human ABHD16A *in vitro* and revealed that it was a lipase with preference for medium-chain and long-chain fatty acids, especially long-chain unsaturated monoglycerides and 15-deoxy-Δ12,14-prostaglandin J2–2-glycerol ester (15d-PGJ2-G) [19], and its esterase hydrolysis activity could effectively be inhibited by hormone-sensitive lipase inhibitors. Another important breakthrough was achieved by Kamat *et al*. [2] who showed that the interplay between ABHD16A and ABHD12 dynamically regulates immunomodulatory lysophosphatidylserines (lyso-PSs) and consequently affects the release of lipopolysaccharide-induced proinflammatory cytokines from macrophages. A previous study showed that homozygous mutations of ABHD12 could cause autosomal recessive neurodegenerative disease characterized by polyneuropathy, hearing loss, ataxia, restenosis pigmentosa and cataract (PHARC) [22–24]. ABHD12 deficiency aggravated neuroinflammation and was closely associated with cerebellar atrophy and peripheral neuroinflammatory disease in cell models, murine PHARC and zebrafish [9,24]. ABHD16A has a higher specific activity with PS than with hydrolysing lysophospholipids, other diacylated phospholipids and neutral lipids [2]. Several selective inhibitors of ABHD16A have been identified through comparative activity-based protein profiling analyses [2,19]. In addition, a more recent study showed that ABHD16A is an immune-balancing regulator that catalyses the hydrolysis of prostaglandin-glycerol (PG-G) in neutrophils [1]. In *Sus scrofa*, a correlation was observed between single nucleotide polymorphisms (SNPs) of the ABHD16A gene and back fat thickness, implying that ABHD16A performs a function in the metabolism of adipose tissue [25].

Several interaction partners of ABHD16A, including IFITM1 (interferon-induced transmembrane proteins), hnRNP1 (heterogeneous nuclear ribonucleoprotein A1), RNF5 (RING zinc-finger domain protein), SPP (signal peptide peptidase), COX II (Mitochondrial cytochrome oxidase II), ATP5G3 (subunit of mitochondrial ATP synthase H^+^ transporter) and NAD3 (Mitochondrial NADH dehydrogenase), were identified through a high-throughput yeast two-hybrid screening [26]. The functions of the interaction proteins above suggest that ABHD16A might play a role in multiple physiological processes. However, no further studies investigating the function or mechanism of these interactions have been reported. A microarray-based long non-coding RNA (lncRNA) expression profiling study showed that the lncRNA of ABHD16A could be a novel biomarker of coronary aneurysm [27]. In activated B cells, the reduced mRNA expression of ABHD16A by MiR-155 provided a clue regarding ABHD16A functions based on the important role played by mic-155 in various physiological and pathological processes [28]. Our latest studies showed that ABHD16A inhibited the application of Japanese encephalitis virus *in vitro* and was associated with the risk of inflammation-related liver disease (JX 2018 unpublished data).

## 6. Research progress related to the ABHD family members

Different members of the ABHD family are located on different chromosomes. These proteins have different numbers of exons and amino acid residues and show expression differences in different tissues. Although the functions of several members are unknown, studies have shown that these proteins play significant roles in glucose and lipid metabolism, immunoregulation and many human diseases (table 2).

**Table 2.** Mammalian ABHD superfamily members. The data regarding the number of exons were obtained from BioGPS, and the data regarding the relatively high expression in normal human tissues were primarily obtained from the BioGPS portal and the reported references.

protein name	molecular weight (kDa)	aliases	gene location in humans	number of exons	relatively high expression in normal human tissues (BioGPS)	related function or role in disease
ABHD1	45	LABH1	2p23.3	9	testis, sperm saphenous	related to oxidative stress in mouse and rat models [29–32]; expression downregulation is driven by hepatic steatosis and insulin resistance induced by Notch signalling [33]
ABHD2	48	HS1–2, LABH2, PHPS1–2	15q26.1	16	prostate, lung, NK cells, whole blood	a glyceridase and ester hydrolase cleaving 2AG and leading to sperm hyperactivation in a progesterone-dependent manner [34–36]; an androgen-regulated gene promoting prostate cancer growth and resistance to chemotherapy [37]; essential for the reproduction of HBV [38,39]; involved in calcium transfer from the endoplasmic reticulum to mitochondria [40] and chronic obstructive pulmonary disease (COPD) in a Chinese Han population [41]; associated with anoikis resistance in ovarian cancer [7] and possibly associated with tumorigenesis in hepatocytes, stomach cells and colon cells [42,43]
ABHD3	46	LABH3	18q11.2	12	colon, small intestine, whole blood	a brain serine hydrolase related to the activation of the endocannabinoid system [44]; a lipase playing the role of a physiological regulator in the metabolism of medium-chain phospholipids [45]; possibly influences innate immunity by transcription factor T-bet [46]
ABHD4	39	ABH4	14q11.2	8	adipocyte, testis	functions in *N*-Acyl ethanolamine synthesis as a (lyso) *N*-acyl phosphatidylethanolamine-selective lipase [47,48]; a novel regulator of anoikis resistance [49]
ABHD5	39	CGI58; IECN2; NCIE2; CDS	3p21.33	8	adipose tissue, bone marrow	a critical acyltransferase with lysophosphatidylglycerol acyltransferase and adipose triglyceride lipase activities and is involved in metabolic disorders; as a lysophosphatidylglycerol acyltransferase, prompts autophagy and is associated with Chanarin-Dorfman syndrome by attenuating inflammatory responsiveness via the promotion of PPAR gamma signalling [50–52]; activates other adipose triglyceride lipases and stimulates triglyceride breakdown as an adipose triglyceride lipase [50,53,54]; a tumour suppressor in human colorectal carcinoma development and progression [55] and serves as a novel tumour marker in sebaceous carcinoma [56]; plays an important role in protecting against atherosclerosis development in macrophages in mice [57]; tissue-specific ABHD5 deficiency leads to lipid imbalance in the liver and plasma caused by the insufficient secretion of postprandial lipoprotein [58], and upregulates gene expression related to hepatic insulin resistance, neutral lipid storage disease, fibrosis, inflammation and hepatic steatosis [59–62]; downregulation of ABHD5 in the heart stimulates the development of diabetic cardiomyopathy by aggravating myocardial steatosis and oxidative stress [63]
ABHD6	38		3p14.3	10	small intestine, spleen, duodenum	as a monoacylglycerol hydrolase, involved in the activation of the endocannabinoid signalling system [44,64–67] and systemic lupus erythematosus [68]; negatively regulates AMPAR-mediated synaptic transmission in hippocampal neurons in HEK293 T cells [69,70]; acts as a critical regulator of metabolic syndrome [71] and energy balance, including the functional realization of brown adipose and the browning of white fat by promoting glucose-stimulated insulin secretion [72]; participates in the pathogenesis of obesity and fatty liver due to its degradation functions in late endosomal/lysosomal lipid Bis [64]; a new potential diagnostic marker or an alternative therapeutic target in Ewing family tumours [73]
ABHD7	42	EPHX4; EH4; EPHXRP	1p22.1	7	brain	a high-activity epoxide hydrolase for fatty acids [74]
ABHD8	47		19p13.11	5	brain	underlying breast and ovarian cancer risk [75]
ABHD9	41	EPHX3; EH3	19p13.12	8	skin, oesophagus	a high-activity epoxide hydrolase for fatty acids [74]; the promoter hypermethylation of ABHD9 possibly leads to prostate cancer recurrence and serves as a marker for prostate cancer prognosis [76,77]
ABHD10	34		3q13.2	6	pineal, kidney, thyroid	affects the formation of immunotoxic metabolites, mycophenolic acid acyl-glucuronide [78,79]; acyl glucuronide and probenecid acyl glucuronide in human liver [80]
ABHD11	35	PP1226; WBSCR21	7q11.23	7	colon, prostate	is associated with the development of distant metastases and serves as a novel biomarker of lung adenocarcinoma [81]
ABHD12	45	PHARC; ABHD12A; BEM46L2; C20orf22; dJ965G21.2	20p11.21	17	thyroid, brain	participates in the breakdown of 2-AG in the central nervous system and along with MAGL and ABHD6, controls 99% of 2-AG hydrolysis in the brain [65,82]; serves as a lysophospholipase and metabolizes lysophosphatidylserine, which participates in the endocannabinoid signalling pathway [9,18]; associated with PHARC [9,22,23,83,84]; a potential indicator of liver diseases in plasma [12]
ABHD12B	41	BEM46L3; C14orf29; c14_5314	14q22.1	15	skin	a gene potentially related to obesity [85], chronic periodontitis [86] and longitudinal changes in ventricle size [87]
ABHD13	39	BEM46L1; C13orf6; bA153I24.2	13q33.3	2	bone marrow, thyroid	very little known
ABHD14A	30	DORZ1	3p21.2	5	kidney, thyroid, adrenal	a candidate gene for autism spectrum disorder [88]; plays a potential role in cerebellar development through Zic1, which is a finger protein that controls vertebrate neural development [89]
ABHD14B	22	CIB; HEL-S-299	3p21.2	4	fat, kidney, liver	a potential structural distinctive cofactor with hydrolase activity for transcription initiation factor [90]; a marker of tumour progression in an unknown primary syndrome in neuroendocrine tumours [91]
ABHD15	52		17q11.2	2	fat	is involved in insulin signalling in adipocytes [92–94]; Plays an important role in the development of adipocytes and apoptosis [95]
ABHD16A	63	BAT5; NG26; PP199; D6S82E	6p21.33	21	testis, brain	refer to the fourth part of the text
ABHD16B	53	C20orf135; dJ591C20.1	20q13.33	1	testis	the methylation of the abhd16b gene was related to COPD [96]
ABHD17A		C19orf27; FAM108A1	19p13.3	7	spleen, bone marrow	as a depalmitoylating enzyme, depalmitoylates Ras [97,98], PSD95 [10,98] and MAP6 [99]
ABHD17B		CGI-67; C9orf77; FAM108B1	9q21.13	8	brain	a depalmitoylating enzyme [10,97–99]
ABHD17C		FAM108C1	15q25.1	3	colon	a depalmitoylating enzyme [10,97–99]
ABHD18	46.9	C4orf29	4q28.2	18	ubiquitously expressed at low to moderate levels	very little is known about this protein; probably associated with human hepatocellular carcinoma (HCC) [100] and fatty acid composition in pig muscle [101]

### 6.1. Direct contribution to lipid metabolism

The biosynthesis and degradation of lipids are vital for organisms to sustain normal life activities, because lipids are important components of cellular structures, sources of energy, intracellular signalling molecules and are also involved in the acyl modification of proteins. The results showed that, in the ABHD protein family, ABHD2, ABHD3, ABHD4, ABHD5, ABHD6, ABHD12 and ABHD16 participated in the metabolism of glycerine esters or phospholipids. ABHD2 is a new triglyceride lipase with an ester hydrolysing capacity [34]. In addition, ABHD2 acted as a progesterone receptor associated with lipid hydrolase in the activation of sperm in the reproductive process [35]. ABHD3 selectively cleaved medium-chain phospholipids and oxidized short phospholipids [45]. In the mammalian nervous system, ABHD4 was a major regulator of *N*-acyl phospholipid metabolism, and had the capacity to hydrolyse *N*-arachidonoyl phosphatidylethanolamine nape, lyso-nape, *N*-acyl-phospholipid serine and other *N*-acyl phospholipids [47,48,102]. ABHD5 could promote the decomposition of triglyceride due to its fatty triglyceride lipase activity [50,53,54]. ABHD6, which is a monoacylglycerol hydrolase, functions in balancing energy, regulating the function of brown adipose and modulating white adipose browning [72]. Both ABHD6 and ABHD12, which are 2-arachidonylglycerol hydrolases, are involved in the endocannabinoid and eicosanoid signalling pathways in the brain [44]. A transcriptome analysis indicated that ABHD18A was probably related to the modulation of fatty acid composition in pig muscle [101].

### 6.2. An important role in liver diseases

The liver is the largest digestive gland and the centre of material and energy metabolism in the human body. As lipases, the ABHD proteins exert significant effects on hepatic glucose and lipid metabolism. The results of one study [59] showed that several members of the ABHD family were related to the occurrence and development of hepatopathy. Liver-specific ABHD5 knockout mice exhibit hepatomegaly and steatosis, and with increasing age the expression of inflammation factors and fibrosis factors at the mRNA level were significantly increased. These results suggest that the deletion of ABHD5 in the liver not only directly leads to liver steatosis but is also involved in steatohepatitis and fibrosis. The mice treated with the ABHD5 antisense oligonucleotide showed severe hepatic steatosis and increased hepatocellular diacylglycerol (DAG), which is a well-documented trigger of insulin resistance, but unexpectedly remained insulin-sensitive [61]. The molecular mechanism could be that a reduction in ABHD5 promotes the isolation of hepatocellular DAG in the lipid droplet/ER section and that the DAG redistribution from the plasma membrane precludes the PKCɛ translocation to the plasma membrane, which leads to liver insulin resistance [61,63]. ABHD18 was identified as a risk factor for liver cirrhosis and HCC because of the genetic variations at loci involved in the immune response [100].

### 6.3. A regulator or marker of certain cancers

Many people worldwide suffer from various cancers, particularly metastatic cancer. Cancer cells have more active motility, stronger drug resistance and a greater tolerance to the host immune system. Cancer cells acquiring anoikis resistance survive after detaching from their primary origin and spreading throughout the body through the circulatory and lymphatic systems. A functional genomics study identified that ABHD2 was a regulator of anoikis resistance in ovarian cancer [7]. The results showed that the silencing of ABHD2 could cause OVCA420 cell apoptosis resistance, and the overexpression of ABHD2 could decrease cell resistance to apoptosis. In addition, the expression of ABHD2 is lower in clinical serous ovarian cancer specimens [7]. Studies have suggested that the expression inhibition of ABHD2 may promote a malignant phenotype and contribute to an adverse prognosis in patients with serous ovarian cancer. ABHD4 is a novel regulator of anoikis sensitivity because ABHD4 knockdown could inhibit anoikis in prostate cells and reduce anoikis sensitivity in nasopharyngeal and ovarian cancer cells, while the overexpression of ABHD4 increased anoikis sensitivity [49]. A deficiency of ABHD5 could promote a shift of metabolism to aerobic glycolysis and contribute to colorectal carcinoma development and progression [55]. Moreover, ABHD5 was identified as a novel reliable marker for distinguishing sebaceous carcinoma from non-sebaceous tumours [56]. Compared with the expression seen in normal tissues, the high expression of ABHD6 in the Ewing sarcoma family of tumours suggested that ABHD6 might be a potential diagnostic marker or drug target [73]. The results from an expression quantitative trait locus (eQTL) analysis showed that the expression of ABHD8 was higher in breast cancer and ovarian cancer than that in normal corresponding organs [75]. Using an activity-based biomarker discovery platform, Wiedl *et al*. identified that ABHD11 is a novel biomarker that, through its activities, could predict the presence of aggressive lung adenocarcinomas and the development of distant metastases [81]. By analysing SNPs and copy number variations in the peripheral blood, Clifford *et al*. [100] found that ABHD18 was an important factor in hepatocellular carcinoma in the Asian population.

### 6.4. A helper or restriction factor in virus infection

Using a human genome-wide bioarray, Ding *et al*. [38] found that ABHD2 could contribute to the proliferation of hepatitis B viruses (HBVs) by analysing the differential expression of HBV-expressing and control cells through a wholegenome expression profiling of hepatitis B. The antisense oligodeoxynucleotides targeting ABHD2 successfully blocked the replication and expression of HBV [38]. Vieyres *et al*. [103] demonstrated that ABHD5 was a new host factor contributing to virus morphogenesis in hepatitis C virus production and could trigger the mobilization and consumption of the luminal lipid droplet, which is important for the envelopment, maturation and budding of infectious virions. Our latest results show that ABHD16A inhibits the proliferation of Japanese encephalitis virus (JX 2018, unpublished data). The finding above suggested that the ABHD proteins might be potential targets in therapies for viral infectious diseases.

### 6.5. A key gene in other diseases

ABHD2 was found to be a critical gene in chronic obstructive pulmonary disease (COPD) by evaluating the genetic variation in the ABHD2 gene among Han Chinese COPD patients and normal controls [41]. The analysis of the DNA methylation data of genes throughout the genome showed that COPD is associated with DNA methylation at the CpG sites of the ABHD16B gene [96]. A mutation of the ABHD5 gene could lead to a rare genetic disorder called Chanarin-Dorfman Syndrome because such patients accumulate excess triacylglycerol caused by a functional defect in ABHD5 in certain tissues and ichthyosis [52,53]. In addition, a reduction in the ABHD5 expression levels in the heart may aggravate myocardial steatosis, oxidative stress and diabetic cardiomyopathy [63]. ABHD6, which is an MAG hydrolase, stimulated insulin secretion induced by glucose in pancreatic beta cells, participated in the regulation of energy homeostasis via PPAR gamma and may represent a new drug target for obesity and type 2 diabetes [72]. Based on genome-wide association studies investigating chronic periodontitis, Rhodin *et al*. [86] found that ABHD12B was associated with chronic periodontitis and worthy of further investigation. ABHD14A was identified as a candidate gene for autism spectrum disorder in an analysis of homozygous haplotype mapping of SNPs [88].

## 7. Conclusion and perspectives

Here, we analysed the gene and protein structure, molecular evolution and existing or presumed functions of ABHD16A, and reviewed the functions of the other ABHD family members. Based on previous findings, we highlight the important roles played by the ABHDs during the occurrence and development of diseases related to lipid metabolism and inflammation.

Human ABHD16A might be a potential diagnostic marker of inflammatory-related diseases or play critical roles in the progress of such diseases. The high conservation of its amino acid sequences, lipase motifs and acyltransferase motifs indicates to a certain extent the necessity of this gene for specific cellular functions in mammalian species. ABHD16A not only participates in lipid metabolism but is also involved in the regulation of inflammation and immunity. Many studies have shown that other members of the ABHD protein family are associated with different diseases, such as cancer, lipid metabolism, liver disease and pulmonary disease. Studies investigating these proteins could not only enhance our understanding of the molecular mechanisms of related illnesses but also contribute to screening novel targets and new drugs. The regulation of virus infection suggested that ABHD could not be ignored as a potential marker or target, especially in an era of emerging and re-emerging viruses that unexpectedly appear.

Therefore, the following are potential future directions: (i) the identification or establishment of the inner link between the ABHD proteins and diseases; (ii) the identification of the enzymatic characteristics of ABHD proteins whose activity remains unknown; (iii) the exploration of the molecular mechanisms or pathways of disease-related ABHD proteins; (iv) the screening of proteins interacting with ABHD, especially in the field of intracellular transport and acylation modification (although ABHD16A and ABHD17 could be involved in the palmitoylation/depalmitoylation cycle, protein transport, organelle localization or special functions [10,104,105], their mechanism and targets must be further studied); and (v) the establishment of model cells or animals. The proper experimental model is crucial for studies investigating diseases. Some techniques (e.g. siRNA or CRISPR/CAS9) have been widely used in studies examining molecular mechanisms, especially of human diseases. Tissue-specific or conditional transgenic mice and gene knockout mice are needed if the offspring have a lower positive rate or the knockout leads to a failure in embryogenesis.

In conclusion, the human ABHD protein family has many members and performs a variety of biological functions. These proteins could play an important role in the regulation of lipid metabolism and signalling transduction pathways, and are possibly directly or indirectly correlated with several human diseases. Although interesting results have been obtained, the functions and molecular mechanisms remain unclear and should be explored in more detail in the future. For researchers, studies in this field could be promising, interesting and significant, especially for understanding several human diseases.

## Data accessibility

This article has no additional data.

## References

[1] Turcotte C, Zarini S, Jean S, Martin C, Murphy RC, Marsolais D, Laviolette M, Blanchet MR, Flamand N 2017 The endocannabinoid metabolite prostaglandin E2 (PGE2)-Glycerol inhibits human neutrophil functions: involvement of its hydrolysis into PGE2 and EP Receptors. J. Immunol. 198, 3255–3263. (doi:10.4049/jimmunol.1601767) 28258202 10.4049/jimmunol.1601767

[2] Kamat SS, Camara K, Parsons WH, Chen DH, Dix MM, Bird TD, Howell AR, Cravatt BF 2015 Immunomodulatory lysophosphatidylserines are regulated by ABHD16A and ABHD12 interplay. Nat. Chem. Biol. 11, 164–171. (doi:10.1038/nchembio.1721) 25580854 10.1038/nchembio.1721 PMC4301979

[3] Hsieh YY, Lin YJ, Chang CC, Chen DY, Hsu CM, Wang YK, Hsu KH, Tsai FJ 2010 Human lymphocyte antigen B-associated transcript 2, 3, and 5 polymorphisms and haplotypes are associated with susceptibility of Kawasaki disease and coronary artery aneurysm. J. Clin. Lab. Anal. 24, 262–268. (doi:10.1002/jcla.20409) 20626023 10.1002/jcla.20409 PMC6647560

[4] Ollis DL et al. 1992 The alpha/beta hydrolase fold. Protein Eng. 5, 197–211. (doi:10.1093/protein/5.3.197) 1409539 10.1093/protein/5.3.197

[5] Nardini M, Dijkstra BW 1999 Alpha/beta hydrolase fold enzymes: the family keeps growing. Curr. Opin Struct. Biol. 9, 732–737. (doi:10.1016/S0959-440X(99)00037-8) 10607665 10.1016/s0959-440x(99)00037-8

[6] Lord CC, Thomas G, Brown JM 2013 Mammalian alpha beta hydrolase domain (ABHD) proteins: lipid metabolizing enzymes at the interface of cell signaling and energy metabolism. Biochim. Biophys. Acta 1831, 792–802. (doi:10.1016/j.bbalip.2013.01.002) 23328280 10.1016/j.bbalip.2013.01.002 PMC4765316

[7] Yamanoi K et al. 2016 Suppression of ABHD2, identified through a functional genomics screen, causes anoikis resistance, chemoresistance and poor prognosis in ovarian cancer. Oncotarget 7, 47 620–47 636. (doi:10.18632/oncotarget.9951) 10.18632/oncotarget.9951 PMC5216966 27323405

[8] Poursharifi P, Madiraju SRM, Prentki M 2017 Monoacylglycerol signalling and ABHD6 in health and disease. Diabetes Obes. Metab. 19(Suppl. 1), 76–89. (doi:10.1111/dom.13008) 28880480 10.1111/dom.13008

[9] Tingaud-Sequeira A et al. 2017 Functional validation of ABHD12 mutations in the neurodegenerative disease PHARC. Neurobiol. Dis. 98, 36–51. (doi:10.1016/j.nbd.2016.11.008) 27890673 10.1016/j.nbd.2016.11.008

[10] Yokoi N, Fukata Y, Sekiya A, Murakami T, Kobayashi K, Fukata M 2016 Identification of PSD-95 depalmitoylating enzymes. J. Neurosci. 36, 6431–6444. (doi:10.1523/JNEUROSCI.0419-16.2016) 27307232 10.1523/JNEUROSCI.0419-16.2016 PMC5015780

[11] Spies T, Bresnahan M, Strominger JL 1989 Human major histocompatibility complex contains a minimum of 19 genes between the complement cluster and HLA-B. Proc. Natl Acad. Sci. USA 86, 8955–8958. (doi:10.1073/pnas.86.22.8955) 2813433 10.1073/pnas.86.22.8955 PMC298409

[12] Spies T, Blanck G, Bresnahan M, Sands J, Strominger JL 1989 A new cluster of genes within the human major histocompatibility complex. Science 243, 214–217. (doi:10.1126/science.2911734) 2911734 10.1126/science.2911734

[13] Sargent CA, Dunham I, Campbell RD 1989 Identification of multiple HTF-island associated genes in the human major histocompatibility complex class III region. EMBO J. 8, 2305–2312. 2477242 10.1002/j.1460-2075.1989.tb08357.x PMC401163

[14] Albertella MR, Jones H, Thomson W, Olavesen MG, Campbell RD 1996 Localization of eight additional genes in the human major histocompatibility complex, including the gene encoding the casein kinase II beta subunit (CSNK2B). Genomics 36, 240–251. (doi:10.1006/geno.1996.0459) 8812450 10.1006/geno.1996.0459

[15] Ikegami H, Kawaguchi Y, Ueda H, Fukuda M, Takakawa K, Fujioka Y, Fujisawa T, Uchida K, Ogihara T 1993 MHC-linked diabetogenic gene of the NOD mouse: molecular mapping of the 3′ boundary of the diabetogenic region. Biochem. Biophys. Res. Commun. 192, 677–682. (doi:10.1006/bbrc.1993.1468) 8097912 10.1006/bbrc.1993.1468

[16] Sanishvili R et al. 2003 Integrating structure, bioinformatics, and enzymology to discover function: BioH, a new carboxylesterase from *Escherichia coli*. J. Biol. Chem. 278, 26 039–26 045. (doi:10.1074/jbc.M303867200) 10.1074/jbc.M303867200 PMC2792009 12732651

[17] Tomczyk NH, Nettleship JE, Baxter RL, Crichton HJ, Webster SP, Campopiano DJ 2002 Purification and characterisation of the BIOH protein from the biotin biosynthetic pathway. FEBS Lett. 513, 299–304. (doi:10.1016/S0014-5793(02)02342-6) 11904168 10.1016/s0014-5793(02)02342-6

[18] Parkkari T et al. 2014 Discovery of triterpenoids as reversible inhibitors of alpha/beta-hydrolase domain containing 12 (ABHD12). PLoS ONE 9, e98286 (doi:10.1371/journal.pone.0098286) 24879289 10.1371/journal.pone.0098286 PMC4045134

[19] Savinainen JR, Patel JZ, Parkkari T, Navia-Paldanius D, Marjamaa JJ, Laitinen T, Nevalainen T, Laitinen JT 2014 Biochemical and pharmacological characterization of the human lymphocyte antigen B-associated transcript 5 (BAT5/ABHD16A). PLoS ONE 9, e109869 (doi:10.1371/journal.pone.0109869) 25290914 10.1371/journal.pone.0109869 PMC4188605

[20] Thomas G, Brown AL, Brown JM 2014 In vivo metabolite profiling as a means to identify uncharacterized lipase function: recent success stories within the alpha beta hydrolase domain (ABHD) enzyme family. Biochim. Biophys. Acta 1841, 1097–1101. (doi:10.1016/j.bbalip.2014.01.004) 24423940 10.1016/j.bbalip.2014.01.004 PMC4069229

[21] Mathew PA, Kumar V, Bennett M, Flaherty L 1995 Identification of a recombinational breakpoint at the BAT5 locus in three intra-H-2 recombinant inbred mouse strains. Exp. Clin. Immunogenet. 12, 261–267. 8919359

[22] Fiskerstrand T et al. 2010 Mutations in ABHD12 cause the neurodegenerative disease PHARC: an inborn error of endocannabinoid metabolism. Am. J. Hum. Genet. 87, 410–417. (doi:10.1016/j.ajhg.2010.08.002) 20797687 10.1016/j.ajhg.2010.08.002 PMC2933347

[23] Chen DH et al. 2013 Two novel mutations in ABHD12: expansion of the mutation spectrum in PHARC and assessment of their functional effects. Hum. Mutat. 34, 1672–1678. (doi:10.1002/humu.22437) 24027063 10.1002/humu.22437 PMC3855015

[24] Blankman JL, Long JZ, Trauger SA, Siuzdak G, Cravatt BF 2013 ABHD12 controls brain lysophosphatidylserine pathways that are deregulated in a murine model of the neurodegenerative disease PHARC. Proc. Natl Acad. Sci. USA 110, 1500–1505. (doi:10.1073/pnas.1217121110) 23297193 10.1073/pnas.1217121110 PMC3557017

[25] Fontanesi L et al. 2012 Identification and association analysis of several hundred single nucleotide polymorphisms within candidate genes for back fat thickness in Italian Large White pigs using a selective genotyping approach. J. Anim. Sci. 90, 2450–2464. (doi:10.2527/jas.2011-4797) 22367074 10.2527/jas.2011-4797

[26] Lehner B, Semple JI, Brown SE, Counsell D, Campbell RD, Sanderson CM 2004 Analysis of a high-throughput yeast two-hybrid system and its use to predict the function of intracellular proteins encoded within the human MHC class III region. Genomics 83, 153–167. (doi:10.1016/S0888-7543(03)00235-0) 14667819 10.1016/s0888-7543(03)00235-0

[27] Cai Y et al. 2016 Circulating ‘lncRNA OTTHUMT00000387022′ from monocytes as a novel biomarker for coronary artery disease. Cardiovasc. Res. 112, 714–724. (doi:10.1093/cvr/cvw022) 26857419 10.1093/cvr/cvw022

[28] Vigorito E et al. 2007 microRNA-155 regulates the generation of immunoglobulin class-switched plasma cells. Immunity 27, 847–859. (doi:10.1016/j.immuni.2007.10.009) 18055230 10.1016/j.immuni.2007.10.009 PMC4135426

[29] van Roon-Mom WM, Pepers BA, 't Hoen PA, Verwijmeren CA, den Dunnen JT, Dorsman JC, van Ommen GB 2008 Mutant huntingtin activates Nrf2-responsive genes and impairs dopamine synthesis in a PC12 model of Huntington's disease. BMC Mol. Biol. 9, 84 (doi:10.1186/1471-2199-9-84) 18844975 10.1186/1471-2199-9-84 PMC2588454

[30] Stoelting M, Geyer M, Reuter S, Reichelt R, Bek MJ, Pavenstadt H 2009 Alpha/beta hydrolase 1 is upregulated in D5 dopamine receptor knockout mice and reduces O2- production of NADPH oxidase. Biochem. Biophys. Res. Commun. 379, 81–85. (doi:10.1016/j.bbrc.2008.12.008) 19073140 10.1016/j.bbrc.2008.12.008

[31] Kohman RA, Rodriguez-Zas SL, Southey BR, Kelley KW, Dantzer R, Rhodes JS 2011 Voluntary wheel running reverses age-induced changes in hippocampal gene expression. PLoS ONE 6, e22654 (doi:10.1371/journal.pone.0022654) 21857943 10.1371/journal.pone.0022654 PMC3152565

[32] Kierstein S, Noyes H, Naessens J, Nakamura Y, Pritchard C, Gibson J, Kemp S, Brass A 2006 Gene expression profiling in a mouse model for African trypanosomiasis. Genes Immun. 7, 667–679. (doi:10.1038/sj.gene.6364345) 17066074 10.1038/sj.gene.6364345 PMC1991335

[33] Fowler JC, Zecchini VR, Jones PH 2011 Intestinal activation of Notch signaling induces rapid onset hepatic steatosis and insulin resistance. PLoS ONE 6, e20767 (doi:10.1371/journal.pone.0020767) 21698231 10.1371/journal.pone.0020767 PMC3116826

[34] Kumar N, Thunuguntla VB, Veeramachaneni GK, Guntupalli S, Bondili JS 2016 Molecular characterization of human ABHD2 as TAG lipase and ester hydrolase. Biosci. Rep. 36, e00358 (doi:10.1042/BSR20160033) 27247428 10.1042/BSR20160033 PMC4945992

[35] Miller MR et al. 2016 Unconventional endocannabinoid signaling governs sperm activation via the sex hormone progesterone. Science 352, 555–559. (doi:10.1126/science.aad6887) 26989199 10.1126/science.aad6887 PMC5373689

[36] Mannowetz N, Miller MR, Lishko PV 2017 Regulation of the sperm calcium channel CatSper by endogenous steroids and plant triterpenoids. Proc. Natl Acad. Sci. USA 114, 5743–5748. (doi:10.1073/pnas.1700367114) 28507119 10.1073/pnas.1700367114 PMC5465908

[37] Obinata D et al. 2016 Abhydrolase domain containing 2, an androgen target gene, promotes prostate cancer cell proliferation and migration. Eur. J. Cancer 57, 39–49. (doi:10.1016/j.ejca.2016.01.002) 26854828 10.1016/j.ejca.2016.01.002

[38] Ding X, Yang J, Wang S 2011 Antisense oligonucleotides targeting abhydrolase domain containing 2 block human hepatitis B virus propagation. Oligonucleotides 21, 77–84. (doi:10.1089/oli.2011.0280) 21466387 10.1089/oli.2011.0280

[39] Ding XR, Yang J, Sun DC, Lou SK, Wang SQ 2008 Whole genome expression profiling of hepatitis B virus-transfected cell line reveals the potential targets of anti-HBV drugs. Pharmacogenomics J 8, 61–70. (doi:10.1038/sj.tpj.6500459) 17505500 10.1038/sj.tpj.6500459

[40] Yun B, Lee H, Powell R, Reisdorph N, Ewing H, Gelb MH, Hsu KL, Cravatt BF, Leslie CC 2017 Regulation of calcium release from the endoplasmic reticulum by the serine hydrolase ABHD2. Biochem. Biophys. Res. Commun. 490, 1226–1231. (doi:10.1016/j.bbrc.2017.06.195) 28684316 10.1016/j.bbrc.2017.06.195 PMC5658124

[41] Liu L, Li X, Yuan R, Zhang H, Qiang L, Shen J, Jin S 2015 Associations of ABHD2 genetic variations with risks for chronic obstructive pulmonary disease in a Chinese Han population. PLoS ONE 10, e0123929 (doi:10.1371/journal.pone.0123929) 25880496 10.1371/journal.pone.0123929 PMC4399978

[42] Yoshida T et al. 2010 Clinical omics analysis of colorectal cancer incorporating copy number aberrations and gene expression data. Cancer Inform. 9, 147–161. (doi:10.4137/CIN.S3851) 20706620 10.4137/cin.s3851 PMC2918356

[43] Chen Y, Guo Y, Ge X, Itoh H, Watanabe A, Fujiwara T, Kodama T, Aburatani H 2006 Elevated expression and potential roles of human Sp5, a member of Sp transcription factor family, in human cancers. Biochem. Biophys. Res. Commun. 340, 758–766. (doi:10.1016/j.bbrc.2005.12.068) 16380080 10.1016/j.bbrc.2005.12.068

[44] Nomura DK, Blankman JL, Simon GM, Fujioka K, Issa RS, Ward AM, Cravatt BF, Casida JE 2008 Activation of the endocannabinoid system by organophosphorus nerve agents. Nat. Chem. Biol. 4, 373–378. (doi:10.1038/nchembio.86) 18438404 10.1038/nchembio.86 PMC2597283

[45] Long JZ, Cisar JS, Milliken D, Niessen S, Wang C, Trauger SA, Siuzdak G, Cravatt BF 2011 Metabolomics annotates ABHD3 as a physiologic regulator of medium-chain phospholipids. Nat. Chem. Biol. 7, 763–765. (doi:10.1038/nchembio.659) 21926997 10.1038/nchembio.659 PMC3201731

[46] Fernandez-Becker NQ, Moss AC 2009 In silico analysis of T-bet activity in peripheral blood mononuclear cells in patients with inflammatory bowel disease (IBD). In Silico Biol. 9, 355–363. (doi:10.3233/ISB-2009-0410) 22430437 10.3233/ISB-2009-0410

[47] Liu J et al. 2008 Multiple pathways involved in the biosynthesis of anandamide. Neuropharmacology 54, 1–7. (doi:10.1016/j.neuropharm.2007.05.020) 17631919 10.1016/j.neuropharm.2007.05.020 PMC2219543

[48] Simon GM, Cravatt BF 2006 Endocannabinoid biosynthesis proceeding through glycerophospho-N-acyl ethanolamine and a role for alpha/beta-hydrolase 4 in this pathway. J. Biol. Chem. 281, 26 465–26 472. (doi:10.1074/jbc.M604660200) 10.1074/jbc.M604660200 16818490

[49] Simpson CD, Hurren R, Kasimer D, MacLean N, Eberhard Y, Ketela T, Moffat J, Schimmer AD 2012 A genome wide shRNA screen identifies alpha/beta hydrolase domain containing 4 (ABHD4) as a novel regulator of anoikis resistance. Apoptosis 17, 666–678. (doi:10.1007/s10495-012-0723-4) 22488300 10.1007/s10495-012-0723-4

[50] Yang D, Chen H, Zeng X, Xie P, Wang X, Liu C 2016 Macrophage CGI-58 attenuates inflammatory responsiveness via promotion of PPARgamma signaling. Cell. Physiol. Biochem. 38, 696–713. (doi:10.1159/000443027) 26872126 10.1159/000443027

[51] Ghosh AK, Ramakrishnan G, Chandramohan C, Rajasekharan R 2008 CGI-58, the causative gene for chanarin-dorfman syndrome, mediates acylation of lysophosphatidic acid. J. Biol. Chem. 283, 24 525–24 533. (doi:10.1074/jbc.M801783200) 10.1074/jbc.M801783200 PMC3259832 18606822

[52] Takeichi T, Sugiura K, Tso S, Simpson MA, McGrath JA, Akiyama M 2016 Bi-allelic nonsense mutations inABHD5 underlie a mild phenotype of dorfman-chanarin syndrome. J. Dermatol. Sci. 81, 134–136. (doi:10.1016/j.jdermsci.2015.10.015) 26547112 10.1016/j.jdermsci.2015.10.015

[53] Lass A et al. 2006 Adipose triglyceride lipase-mediated lipolysis of cellular fat stores is activated by CGI-58 and defective in Chanarin-Dorfman syndrome. Cell Metab. 3, 309–319. (doi:10.1016/j.cmet.2006.03.005) 16679289 10.1016/j.cmet.2006.03.005

[54] Dettlaff-Pokora A, Sledzinski T, Swierczynski J 2016 Upregulation of Pnpla2 and Abhd5 and downregulation of G0s2 gene expression in mesenteric white adipose tissue as a potential reason for elevated concentration of circulating NEFA after removal of retroperitoneal, epididymal, and inguinal adipose tissue. Mol. Cell. Biochem. 422, 21–29. (doi:10.1007/s11010-016-2800-4) 27590244 10.1007/s11010-016-2800-4 PMC5055569

[55] Ou J et al. 2014 Loss of abhd5 promotes colorectal tumor development and progression by inducing aerobic glycolysis and epithelial-mesenchymal transition. Cell Rep. 9, 1798–1811. (doi:10.1016/j.celrep.2014.11.016) 25482557 10.1016/j.celrep.2014.11.016 PMC4268306

[56] Chen WS, Chen PL, Li J, Lind AC, Lu D 2013 Lipid synthesis and processing proteins ABHD5, PGRMC1 and squalene synthase can serve as novel immunohistochemical markers for sebaceous neoplasms and differentiate sebaceous carcinoma from sebaceoma and basal cell carcinoma with clear cell features. J. Cutan. Pathol. 40, 631–638. (doi:10.1111/cup.12147) 23557589 10.1111/cup.12147

[57] Xie P, Zeng X, Xiao J, Sun B, Yang D 2014 Transgenic CGI-58 expression in macrophages alleviates the atherosclerotic lesion development in ApoE knockout mice. Biochim. Biophys. Acta 1841, 1683–1690. (doi:10.1016/j.bbalip.2014.08.014) 25178844 10.1016/j.bbalip.2014.08.014

[58] Xie P, Guo F, Ma Y, Zhu H, Wang F, Xue B, Shi H, Yang J, Yu L 2014 Intestinal Cgi-58 deficiency reduces postprandial lipid absorption. PLoS ONE 9, e91652 (doi:10.1371/journal.pone.0091652) 24618586 10.1371/journal.pone.0091652 PMC3950255

[59] Guo F et al. 2013 Deficiency of liver comparative gene identification-58 causes steatohepatitis and fibrosis in mice. J. Lipid Res. 54, 2109–2120. (doi:10.1194/jlr.M035519) 23733885 10.1194/jlr.M035519 PMC3708361

[60] Lord CC, Brown JM 2012 Distinct roles for alpha-beta hydrolase domain 5 (ABHD5/CGI-58) and adipose triglyceride lipase (ATGL/PNPLA2) in lipid metabolism and signaling. Adipocyte 1, 123–131. (doi:10.4161/adip.20035) 23145367 10.4161/adip.20035 PMC3492958

[61] Cantley JL et al. 2013 CGI-58 knockdown sequesters diacylglycerols in lipid droplets/ER-preventing diacylglycerol-mediated hepatic insulin resistance. Proc. Natl Acad. Sci. USA 110, 1869–1874. (doi:10.1073/pnas.1219456110) 23302688 10.1073/pnas.1219456110 PMC3562813

[62] Schweiger M, Lass A, Zimmermann R, Eichmann TO, Zechner R 2009 Neutral lipid storage disease: genetic disorders caused by mutations in adipose triglyceride lipase/PNPLA2 or CGI-58/ABHD5. Am. J. Physiol. Endocrinol. Metab. 297, E289–E296. (doi:10.1152/ajpendo.00099.2009) 19401457 10.1152/ajpendo.00099.2009

[63] Inoue T et al. 2013 Downregulation of adipose triglyceride lipase in the heart aggravates diabetic cardiomyopathy in db/db mice. Biochem. Biophys. Res. Commun. 438, 224–229. (doi:10.1016/j.bbrc.2013.07.063) 23886955 10.1016/j.bbrc.2013.07.063

[64] Pribasnig MA et al. 2015 alpha/beta hydrolase domain-containing 6 (ABHD6) degrades the late endosomal/lysosomal lipid bis(monoacylglycero)phosphate. J. Biol. Chem. 290, 29 869–29 881. (doi:10.1074/jbc.M115.669168) 10.1074/jbc.M115.669168 PMC4705992 26491015

[65] Savinainen JR, Saario SM, Laitinen JT 2012 The serine hydrolases MAGL, ABHD6 and ABHD12 as guardians of 2-arachidonoylglycerol signalling through cannabinoid receptors. Acta Physiol. 204, 267–276. (doi:10.1111/j.1748-1716.2011.02280.x) 10.1111/j.1748-1716.2011.02280.x PMC3320662 21418147

[66] Marrs WR, Horne EA, Ortega-Gutierrez S, Cisneros JA, Xu C, Lin YH, Muccioli GG, Lopez-Rodriguez ML, Stella N 2011 Dual inhibition of alpha/beta-hydrolase domain 6 and fatty acid amide hydrolase increases endocannabinoid levels in neurons. J. Biol. Chem. 286, 28 723–28 728. (doi:10.1074/jbc.M110.202853) 10.1074/jbc.M110.202853 PMC3190680 21665953

[67] Marrs WR et al. 2010 The serine hydrolase ABHD6 controls the accumulation and efficacy of 2-AG at cannabinoid receptors. Nat. Neurosci. 13, 951–957. (doi:10.1038/nn.2601) 20657592 10.1038/nn.2601 PMC2970523

[68] Oparina NY et al. 2015 PXK locus in systemic lupus erythematosus: fine mapping and functional analysis reveals novel susceptibility gene ABHD6. Ann. Rheum. Dis. 74, e14 (doi:10.1136/annrheumdis-2013-204909) 24534757 10.1136/annrheumdis-2013-204909

[69] Wei M et al. 2016 alpha/beta-Hydrolase domain-containing 6 (ABHD6) negatively regulates the surface delivery and synaptic function of AMPA receptors. Proc. Natl Acad. Sci. USA 113, E2695–E2704. (doi:10.1073/pnas.1524589113) 27114538 10.1073/pnas.1524589113 PMC4868484

[70] Wei M et al. 2017 The inhibitory effect of alpha/beta-hydrolase domain-containing 6 (ABHD6) on the surface targeting of GluA2- and GluA3-Containing AMPA Receptors. Front. Mol. Neurosci. 10, 55 (doi:10.3389/fnmol.2017.00055) 28303090 10.3389/fnmol.2017.00055 PMC5333494

[71] Thomas G et al. 2013 The serine hydrolase ABHD6 Is a critical regulator of the metabolic syndrome. Cell Rep. 5, 508–520. (doi:10.1016/j.celrep.2013.08.047) 24095738 10.1016/j.celrep.2013.08.047 PMC3833083

[72] Zhao S et al. 2016 alpha/beta-Hydrolase domain 6 deletion induces adipose browning and prevents obesity and Type 2 diabetes. Cell Rep. 14, 2872–2888. (doi:10.1016/j.celrep.2016.02.076) 26997277 10.1016/j.celrep.2016.02.076

[73] Max D, Hesse M, Volkmer I, Staege MS 2009 High expression of the evolutionarily conserved alpha/beta hydrolase domain containing 6 (ABHD6) in Ewing tumors. Cancer Sci. 100, 2383–2389. (doi:10.1111/j.1349-7006.2009.01347.x) 19793082 10.1111/j.1349-7006.2009.01347.x PMC11158961

[74] Decker M et al. 2012 EH3 (ABHD9): the first member of a new epoxide hydrolase family with high activity for fatty acid epoxides. J. Lipid Res. 53, 2038–2045. (doi:10.1194/jlr.M024448) 22798687 10.1194/jlr.M024448 PMC3435537

[75] Lawrenson K et al. 2016 Functional mechanisms underlying pleiotropic risk alleles at the 19p13.1 breast-ovarian cancer susceptibility locus. Nat. Commun. 7, 12675 (doi:10.1038/ncomms12675) 27601076 10.1038/ncomms12675 PMC5023955

[76] Cottrell S et al. 2007 Discovery and validation of 3 novel DNA methylation markers of prostate cancer prognosis. J. Urol. 177, 1753–1758. (doi:10.1016/j.juro.2007.01.010) 17437806 10.1016/j.juro.2007.01.010

[77] Stott-Miller M et al. 2014 Validation study of genes with hypermethylated promoter regions associated with prostate cancer recurrence. Cancer Epidemiol. Biomarkers Prev. 23, 1331–1339. (doi:10.1158/1055-9965.EPI-13-1000) 24718283 10.1158/1055-9965.EPI-13-1000 PMC4082437

[78] Iwamura A, Fukami T, Higuchi R, Nakajima M, Yokoi T 2012 Human alpha/beta hydrolase domain containing 10 (ABHD10) is responsible enzyme for deglucuronidation of mycophenolic acid acyl-glucuronide in liver. J. Biol. Chem. 287, 9240–9249. (doi:10.1074/jbc.M111.271288) 22294686 10.1074/jbc.M111.271288 PMC3308823

[79] Fukami T, Yokoi T 2012 The emerging role of human esterases. Drug Metab. Pharmacokinet 27, 466–477. (doi:10.2133/dmpk.DMPK-12-RV-042) 22813719 10.2133/dmpk.dmpk-12-rv-042

[80] Ito Y, Fukami T, Yokoi T, Nakajima M 2014 An orphan esterase ABHD10 modulates probenecid acyl glucuronidation in human liver. Drug Metab. Dispos. 42, 2109–2116. (doi:10.1124/dmd.114.059485) 25217485 10.1124/dmd.114.059485

[81] Wiedl T, Arni S, Roschitzki B, Grossmann J, Collaud S, Soltermann A, Hillinger S, Aebersold R, Weder W 2011 Activity-based proteomics: identification of ABHD11 and ESD activities as potential biomarkers for human lung adenocarcinoma. J. Proteomics 74, 1884–1894. (doi:10.1016/j.jprot.2011.04.030) 21596165 10.1016/j.jprot.2011.04.030

[82] Navia-Paldanius D, Savinainen JR, Laitinen JT 2012 Biochemical and pharmacological characterization of human alpha/beta-hydrolase domain containing 6 (ABHD6) and 12 (ABHD12). J. Lipid Res. 53, 2413–2424. (doi:10.1194/jlr.M030411) 22969151 10.1194/jlr.M030411 PMC3466009

[83] Yoshimura H, Hashimoto T, Murata T, Fukushima K, Sugaya A, Nishio SY, Usami S 2015 Novel ABHD12 mutations in PHARC patients: the differential diagnosis of deaf-blindness. Ann. Otol. Rhinol. Laryngol. 124(Suppl. 1), 77S–83S. (doi:10.1177/0003489415574513) 25743180 10.1177/0003489415574513

[84] Lerat J, Cintas P, Beauvais-Dzugan H, Magdelaine C, Sturtz F, Lia AS 2017 A complex homozygous mutation in ABHD12 responsible for PHARC syndrome discovered with NGS and review of the literature. J. Peripher. Nerv. Syst. 22, 77–84. (doi:10.1111/jns.12216) 28448692 10.1111/jns.12216

[85] Kogelman LJ, Zhernakova DV, Westra HJ, Cirera S, Fredholm M, Franke L, Kadarmideen HN 2015 An integrative systems genetics approach reveals potential causal genes and pathways related to obesity. Genome Med. 7, 105 (doi:10.1186/s13073-015-0229-0) 26482556 10.1186/s13073-015-0229-0 PMC4617184

[86] Rhodin K, Divaris K, North KE, Barros SP, Moss K, Beck JD, Offenbacher S 2014 Chronic periodontitis genome-wide association studies: gene-centric and gene set enrichment analyses. J. Dent. Res. 93, 882–890. (doi:10.1177/0022034514544506) 25056994 10.1177/0022034514544506 PMC4213253

[87] Koran ME, Hohman TJ, Meda SA, Thornton-Wells TA 2014 Genetic interactions within inositol-related pathways are associated with longitudinal changes in ventricle size. J. Alzheimers Dis. 38, 145–154. (doi:10.3233/JAD-130989) 24077433 10.3233/JAD-130989 PMC3815519

[88] Casey JP et al. 2012 A novel approach of homozygous haplotype sharing identifies candidate genes in autism spectrum disorder. Hum. Genet. 131, 565–579. (doi:10.1007/s00439-011-1094-6) 21996756 10.1007/s00439-011-1094-6 PMC3303079

[89] Hoshino J, Aruga J, Ishiguro A, Mikoshiba K 2003 Dorz1, a novel gene expressed in differentiating cerebellar granule neurons, is down-regulated in Zic1-deficient mouse. Brain Res. Mol. Brain Res. 120, 57–64. (doi:10.1016/j.molbrainres.2003.10.004) 14667578 10.1016/j.molbrainres.2003.10.004

[90] Padmanabhan B, Kuzuhara T, Adachi N, Horikoshi M 2004 The crystal structure of CCG1/TAF(II)250-interacting factor B (CIB). J. Biol. Chem. 279, 9615–9624. (doi:10.1074/jbc.M312165200) 14672934 10.1074/jbc.M312165200

[91] Posorski N, Kaemmerer D, Ernst G, Grabowski P, Hoersch D, Hommann M, von Eggeling F 2011 Localization of sporadic neuroendocrine tumors by gene expression analysis of their metastases. Clin. Exp. Metastasis 28, 637–647. (doi:10.1007/s10585-011-9397-5) 21681495 10.1007/s10585-011-9397-5

[92] Gridley S, Lane WS, Garner CW, Lienhard GE 2005 Novel insulin-elicited phosphoproteins in adipocytes. Cell. Signal. 17, 59–66. (doi:10.1016/j.cellsig.2004.05.013) 15451025 10.1016/j.cellsig.2004.05.013

[93] Chavez JA, Gridley S, Sano H, Lane WS, Lienhard GE 2006 The 47 kDa Akt substrate associates with phosphodiesterase 3B and regulates its level in adipocytes. Biochem. Biophys. Res. Commun. 342, 1218–1222. (doi:10.1016/j.bbrc.2006.02.091) 16516160 10.1016/j.bbrc.2006.02.091

[94] Omar B, Zmuda-Trzebiatowska E, Manganiello V, Goransson O, Degerman E 2009 Regulation of AMP-activated protein kinase by cAMP in adipocytes: roles for phosphodiesterases, protein kinase B, protein kinase A, Epac and lipolysis. Cell. Signal. 21, 760–766. (doi:10.1016/j.cellsig.2009.01.015) 19167487 10.1016/j.cellsig.2009.01.015 PMC3576575

[95] Walenta E et al. 2013 alpha/beta-hydrolase domain containing protein 15 (ABHD15)--an adipogenic protein protecting from apoptosis. PLoS ONE 8, e79134 (doi:10.1371/journal.pone.0079134) 24236098 10.1371/journal.pone.0079134 PMC3827343

[96] Wan ES et al. 2012 Systemic steroid exposure is associated with differential methylation in chronic obstructive pulmonary disease. Am. J. Respir. Crit. Care Med. 186, 1248–1255. (doi:10.1164/rccm.201207-1280OC) 23065012 10.1164/rccm.201207-1280OC PMC3622442

[97] Lin DTS, Davis NG, Conibear E 2017 Targeting the Ras palmitoylation/depalmitoylation cycle in cancer. Biochem. Soc. Trans. 45, 913–921. (doi:10.1042/BST20160303) 28630138 10.1042/BST20160303

[98] Lin DT, Conibear E 2015 ABHD17 proteins are novel protein depalmitoylases that regulate N-Ras palmitate turnover and subcellular localization. eLife 4, e11306 (doi:10.7554/eLife.11306) 26701913 10.7554/eLife.11306 PMC4755737

[99] Tortosa E, Adolfs Y, Fukata M, Pasterkamp RJ, Kapitein LC, Hoogenraad CC 2017 Dynamic palmitoylation targets MAP6 to the axon to promote microtubule stabilization during neuronal polarization. Neuron 94, 809–825.e807. (doi:10.1016/j.neuron.2017.04.042) 28521134 10.1016/j.neuron.2017.04.042

[100] Clifford RJ et al. 2010 Genetic variations at loci involved in the immune response are risk factors for hepatocellular carcinoma. Hepatology 52, 2034–2043. (doi:10.1002/hep.23943) 21105107 10.1002/hep.23943 PMC8259333

[101] Puig-Oliveras A, Ramayo-Caldas Y, Corominas J, Estelle J, Perez-Montarelo D, Hudson NJ, Casellas J, Folch JM, Ballester M 2014 Differences in muscle transcriptome among pigs phenotypically extreme for fatty acid composition. PLoS ONE 9, e99720 (doi:10.1371/journal.pone.0099720) 24926690 10.1371/journal.pone.0099720 PMC4057286

[102] Lee HC, Simon GM, Cravatt BF 2015 ABHD4 regulates multiple classes of N-acyl phospholipids in the mammalian central nervous system. Biochemistry 54, 2539–2549. (doi:10.1021/acs.biochem.5b00207) 25853435 10.1021/acs.biochem.5b00207 PMC4767004

[103] Vieyres G, Welsch K, Gerold G, Gentzsch J, Kahl S, Vondran FW, Kaderali L, Pietschmann T 2016 ABHD5/CGI-58, the chanarin-dorfman syndrome protein, mobilises lipid stores for hepatitis C virus production. PLoS Pathog. 12, e1005568 (doi:10.1371/journal.ppat.1005568) 27124600 10.1371/journal.ppat.1005568 PMC4849665

[104] Fukata Y, Murakami T, Yokoi N, Fukata M 2016 Local palmitoylation cycles and specialized membrane domain organization. Curr. Top. Membr. 77, 97–141. (doi:10.1016/bs.ctm.2015.10.003) 26781831 10.1016/bs.ctm.2015.10.003

[105] Martin BR, Cravatt BF 2009 Large-scale profiling of protein palmitoylation in mammalian cells. Nat. Methods 6, 135–138. (doi:10.1038/nmeth.1293) 19137006 10.1038/nmeth.1293 PMC2775068

